# Genomic Risk Prediction of Coronary Artery Disease in 480,000 Adults: Implications for Primary Prevention

**DOI:** 10.1016/j.jacc.2018.07.079

**Published:** 2018-10-16

**Authors:** Michael Inouye, Gad Abraham, Christopher P. Nelson, Angela M. Wood, Michael J. Sweeting, Frank Dudbridge, Florence Y. Lai, Stephen Kaptoge, Marta Brozynska, Tingting Wang, Shu Ye, Thomas R. Webb, Martin K. Rutter, Ioanna Tzoulaki, Riyaz S. Patel, Ruth J.F. Loos, Bernard Keavney, Harry Hemingway, John Thompson, Hugh Watkins, Panos Deloukas, Emanuele Di Angelantonio, Adam S. Butterworth, John Danesh, Nilesh J. Samani

**Affiliations:** a Cambridge Baker Systems Genomics Initiative, Melbourne, Victoria, Australia, and Cambridge, United Kingdom; b Baker Heart and Diabetes Institute, Melbourne, Victoria, Australia; c MRC/BHF Cardiovascular Epidemiology Unit, Department of Public Health and Primary Care, University of Cambridge, Cambridge, United Kingdom; d Department of Clinical Pathology and School of BioSciences, University of Melbourne, Parkville, Victoria, Australia; e The Alan Turing Institute, London, United Kingdom; f Department of Cardiovascular Sciences and NIHR Leicester Biomedical Centre, University of Leicester, Leicester, United Kingdom; g Department of Health Sciences, University of Leicester, Leicester, United Kingdom; h National Institute for Health Research Blood and Transplant Research Unit (NIHR BTRU) in Donor Health and Genomics at the University of Cambridge, Cambridge, United Kingdom; i Division of Diabetes, Endocrinology and Gastroenterology, School of Medical Sciences, Faculty of Biology, Medicine and Health, University of Manchester, Manchester Academic Health Science Centre, Manchester, United Kingdom; j Manchester Diabetes Centre, Manchester University NHS Foundation Trust, Manchester Academic Health Science Centre, Manchester, United Kingdom; k Department of Epidemiology and Biostatistics, Imperial College London, London, United Kingdom; l Department of Hygiene and Epidemiology, University of Ioannina, Ioannina, Greece; m Institute of Cardiovascular Sciences, University College London, London, United Kingdom; n Barts Heart Centre, St. Bartholomew's Hospital, London, United Kingdom; o Charles Bronfman Institute for Personalized Medicine, Mindich Child Health and Development Institute, Icahn School of Medicine at Mount Sinai, New York, New York; p Division of Cardiovascular Sciences, School of Medical Sciences, Faculty of Biology, Medicine and Health, University of Manchester, Manchester, United Kingdom; q Manchester University NHS Foundation Trust, Manchester Academic Health Science Centre, Manchester, United Kingdom; r The Farr Institute of Health Informatics Research and the National Institute for Health Research, Biomedical Research Centre, University College London, London, United Kingdom; s Division of Cardiovascular Medicine, Radcliffe Department of Medicine, University of Oxford, Oxford, United Kingdom; t The Wellcome Trust Centre for Human Genetics, University of Oxford, Oxford, United Kingdom; u William Harvey Research Institute, Barts and the London School of Medicine and Dentistry, Queen Mary University of London, London, United Kingdom; v Wellcome Trust Sanger Institute, Wellcome Genome Campus, Hinxton, Cambridgeshire, United Kingdom

**Keywords:** coronary artery disease, genomic risk prediction, primary prevention, BMI, body mass index, CAD, coronary artery disease, CI, confidence interval, GRS, genomic risk score(s), HR, hazard ratio

## Abstract

**Background:**

Coronary artery disease (CAD) has substantial heritability and a polygenic architecture. However, the potential of genomic risk scores to help predict CAD outcomes has not been evaluated comprehensively, because available studies have involved limited genomic scope and limited sample sizes.

**Objectives:**

This study sought to construct a genomic risk score for CAD and to estimate its potential as a screening tool for primary prevention.

**Methods:**

Using a meta-analytic approach to combine large-scale, genome-wide, and targeted genetic association data, we developed a new genomic risk score for CAD (metaGRS) consisting of 1.7 million genetic variants. We externally tested metaGRS, both by itself and in combination with available data on conventional risk factors, in 22,242 CAD cases and 460,387 noncases from the UK Biobank.

**Results:**

The hazard ratio (HR) for CAD was 1.71 (95% confidence interval [CI]: 1.68 to 1.73) per SD increase in metaGRS, an association larger than any other externally tested genetic risk score previously published. The metaGRS stratified individuals into significantly different life course trajectories of CAD risk, with those in the top 20% of metaGRS distribution having an HR of 4.17 (95% CI: 3.97 to 4.38) compared with those in the bottom 20%. The corresponding HR was 2.83 (95% CI: 2.61 to 3.07) among individuals on lipid-lowering or antihypertensive medications. The metaGRS had a higher C-index (C = 0.623; 95% CI: 0.615 to 0.631) for incident CAD than any of 6 conventional factors (smoking, diabetes, hypertension, body mass index, self-reported high cholesterol, and family history). For men in the top 20% of metaGRS with >2 conventional factors, 10% cumulative risk of CAD was reached by 48 years of age.

**Conclusions:**

The genomic score developed and evaluated here substantially advances the concept of using genomic information to stratify individuals with different trajectories of CAD risk and highlights the potential for genomic screening in early life to complement conventional risk prediction.

As coronary artery disease (CAD) is the leading cause of morbidity and mortality worldwide, early identification of individuals who are at high risk of CAD is essential for primary prevention. As the heritability of CAD has been estimated to be 40% to 60%, comprehensive information on genetic susceptibility could contribute importantly to CAD risk stratification 1, 2. Although family history has long been identified as a risk factor for CAD, elucidation of the genetic architecture of CAD has advanced substantially only during the past decade with the advent of genome-wide association studies. Results from these assumption-free surveys across the genome have laid foundations for developing genomic risk scores (GRS) in the estimation of an individual's underlying genomic risk 3, 4, 5, 6, 7, 8, 9. Furthermore, because GRS are based on germline DNA, they are quantifiable in early life, at or before birth. Hence, they offer the potential for early risk screening and primary prevention before other conventional risk factors become informative.

Due to several inter-related factors, however, previous GRS for CAD have been unable to provide comprehensive assessment of the potential of using genomic information in CAD risk prediction. First, because previously published GRS have utilized only genetic variants of genome-wide significance 4, 5, 8 or involved genotyping arrays that focused only on pre-selected loci (3), they have not fully utilized genome-wide variation, preventing accurate estimation of the relative contribution of each genetic variant to CAD risk. Second, because previous studies of GRS have tended to have moderate statistical power, they have been unable to provide precise effect size estimates 10, 11, 12. Third, because previous studies of GRS have largely lacked external testing in large-scale cohorts that represent a diversity of ancestries (3) and typically have involved only a narrow spectrum of CAD burden (e.g., inclusion of myocardial infarction only) 13, 14, their generalizability has been limited.

Here, we report a more powerful and generalizable genome-wide GRS for CAD to provide a more comprehensive evaluation. We utilized a meta-analytic strategy to construct a GRS for CAD (metaGRS) that captures the totality of information from the largest previous genome-wide association studies, and then investigated the external performance of this metaGRS in stratifying CAD risk in >480,000 individuals from the UK Biobank (UKB) (15). Furthermore, we assessed the effects of 6 conventional risk factors (smoking, blood pressure, body mass index [BMI], diabetes, family history, and high cholesterol) on different genomic risk backgrounds, with the aim of delineating event rates across age, sex, clinical risk factors, and genomic risk score strata to identify individuals who are more likely to benefit from earlier and more intensive therapies. Finally, to assess the potential therapeutic implications of genomic risk scores, we tested the impact of blood pressure and lipid-lowering medication on the performance of the metaGRS.

## Methods

### Study design and participants

The design of this study is shown in Online Figure 1. Details of the design of the UKB have been reported previously (15). Participants were members of the general U.K. population between age 40 and 69 years at recruitment, identified through primary care lists, who accepted an invitation to attend 1 of the 22 assessment centers that were serially established across the United Kingdom between 2006 and 2010. At recruitment, detailed information was collected via a standardized questionnaire on sociodemographic characteristics, health status and physician-diagnosed medical conditions, family history, and lifestyle factors. Selected physical and functional measurements were obtained, including height, weight, waist-hip ratio, and systolic and diastolic blood pressures. The UKB data were subsequently linked to Hospital Episode Statistics (HES) data, as well as national death and cancer registries. The HES data available for the current analysis cover all hospital admissions to NHS hospitals in England and Scotland from April 1997 to March 2015, with the Scottish data dating back as early as 1981. HES uses International Classification of Diseases (ICD)–9th and 10th Revisions to record diagnosis information, and OPCS-4 (Office of Population, Censuses and Surveys: Classification of Interventions and Procedures, version 4) to code operative procedures. Death registries include all deaths in the United Kingdom until January 2016, with both primary and contributory causes of death coded in ICD-10.

CAD was defined as fatal or nonfatal myocardial infarction (MI) cases, percutaneous transluminal coronary angioplasty (PTCA), or coronary artery bypass grafting (CABG). The age of event in prevalent cases was determined by self-reported age and calculated age based on the earliest hospital record for the event; if both self-reported age and calculated age were available, the smaller value was used. For incident cases, hospital and/or death records were used to determined age of event. Prevalent versus incident status was relative to the UKB enrollment assessment. In UKB self-reported data, cases were defined as having had a heart attack diagnosed by a doctor (data field #6150); “non-cancer illnesses that self-reported as heart attack” (data field #20002); or self-reported operation including PTCA, CABG, or triple heart bypass (data field #20004). In HES hospital episodes data and death registry data, MI was defined as hospital admission or cause of death due to ICD-9 410 to 412, or ICD-10 I21 to I24 or I25.2; CABG and PTCA were defined as hospital admission OPCS-4 K40 to K46, K49, K50.1, or K75.

We defined risk factors at the first assessment as follows: diabetes diagnosed by a doctor (field #2443), BMI (field #21001), current smoking (field #20116), hypertension, family history of heart disease, and high cholesterol. For hypertension we used an expanded definition including self-reported high blood pressure (either on blood pressure medication, data fields #6177, #6153; systolic blood pressure >140 mm Hg, fields #4080, #93; or diastolic blood pressure >90 mm Hg, data fields #4079, #94). For family history of heart disease, we considered history in any first-degree relative (father, mother, sibling; fields #20107, 20110, and 20111, respectively). For high cholesterol, we considered individuals with self-reported high cholesterol at assessment, as well as diagnoses in the HES/death records (ICD-9 272.0; ICD-10 E78.0). For the analyses of the number of elevated risk factors, we considered diagnosed diabetes (yes/no), hypertension at assessment (yes/no), BMI >30 kg/m^2^, smoking at assessment (yes/no), high cholesterol (yes/no), and family history of heart disease (yes/no).

Genotyping of UK Biobank participants was undertaken using a custom-built genome-wide array (the UK Biobank Axiom array) of ∼826,000 markers. Genotyping was done in 2 phases. A total of 50,000 subjects were initially typed as part of the UK BiLEVE project (16). The rest of the participants were genotyped using a slightly modified array. Imputation to ∼92 million markers was subsequently carried out using the Haplotype Reference Consortium (17) and UK10K/1000Genomes haplotype resource panels; however, at the time of analysis, known issues existed with the imputation using the latter panel.

### Data processing and quality control

A detailed description is available in the Online Appendix. Briefly, we adapted appropriate quality-control procedures to the set of GWAS (genome-wide association study) summary statistics being utilized, filtering genetic variants for minor allele frequency, Hardy-Weinberg equilibrium, and imputation quality using PLINK (18). Population structure was controlled using the genetic principal components (PCs) supplied by UKB (16). Individuals from UKB were removed if they were diagnosed with coronary aneurysm or had no CAD event date information.

### Construction of the metaGRS

A detailed description is available in the Online Appendix. Briefly, we built a meta-score (metaGRS) based on 3 genetic risk scores: 1) a previously published score (GRS46K) of 46,000 SNPs derived from a genetic association study using Metabochip, a genotyping array with a focus on cardiometabolic genetic loci (3); 2) a score of 202 genetic variants significantly associated with CAD at false discovery rate <0.05 (FDR202) in a recent GWAS from CARDIoGRAMplusC4D (18); and 3) a genome-wide polygenic score (1000Genomes) based on the same GWAS (18). To derive the 1000Genomes score and weight the 3 genetic risk scores for the metaGRS, we used a small training set from UKB (n = 3,000 individuals). The remaining 482,629 UKB individuals not in the training set comprised the external validation set.

### Statistical analysis

All scores were standardized to zero-mean and unit-variance. All scores were evaluated using logistic regression or age-as-time-scale Cox proportional hazards regression, with censoring at 75 years, as well as with Kaplan-Meier estimates of cumulative incidence (censored at 75 years). Unless otherwise noted, analyses using only genetic risk scores include both prevalent and incident CAD cases (germline DNA variation being determined prior to any disease); to avoid reverse causation, analyses that included conventional risk factors (measured at the UKB assessment) used only incident CAD. The Cox models were stratified by sex and adjusted for genotyping array (BiLEVE vs. UKB) and 10 genetic PCs. C-indexes for the Cox models were sex stratified, using age as the time scale. A competing risk analysis, using the Aalen-Johansen estimator (3 states: CAD, non-CAD death, and censored), was conducted using the R package “survival” version 2.41-3 (R Foundation for Statistical Computing, Vienna, Austria) (19). The precision-recall curves (equivalent to the positive-predictive-value vs sensitivity curve) were computed in the R package “ROCR” (20), and the area under the curve was computed using numerical integration.

## Results

**Figure 1:**
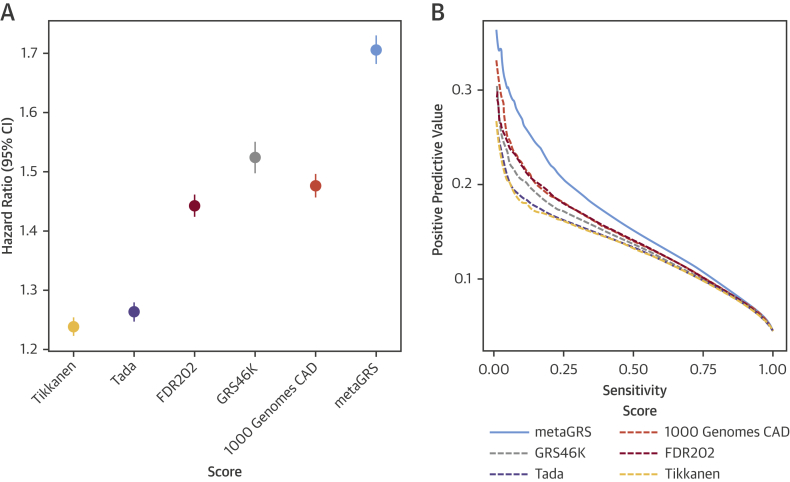
Relative Performance of Individual Genomic Risk Scores for CAD Compared With the metaGRS In the UKB validation set (n = 482,629), **(A)** hazard ratios per SD of each score for all CAD (n = 22,242), censored at age 75 years, from Cox regression stratified by sex and adjusted for genotyping array (BiLEVE/UKB) and 10 genetic PCs. **(B)** Positive predictive value versus sensitivity for a logistic regression for each GRS, adjusted for sex, age, genotyping array (BiLEVE/UKB), and 10 genetic PCs. CAD = coronary artery disease; CI = confidence interval; GRS = genomic risk score(s); PCs = principal components.

The characteristics of the UKB subjects in the external validation set (N = 482,629) are shown in Table 1, comprising 22,242 CAD cases before age 75 years and 460,387 noncases in total. There were 9,729 prevalent cases of CAD at the time of recruitment, and a further 12,513 incident cases of CAD during a mean follow-up of 6.2 years, at the censoring age of 75 years in 2017. Our meta-analysis approach resulted in a “metaGRS” comprising 1,745,180 genetic variants, themselves explaining 26.8% of CAD heritability (Online Appendix). A comparison of the metaGRS with its individual components and previously published GRS from Tikkanen et al. (6) and Tada et al. (8) in the UKB external validation set is given in Figure 1, showing that the metaGRS had substantially greater association with CAD risk in terms of hazard ratio (HR) as well as positive predictive value at any given sensitivity.

**Table 1:** Study Characteristics

	UK Biobank (N = 482,629)	Male (n = 220,284) (45.6%)	Female (n = 262,345) (54.4%)
Age at assessment, yrs	56.5 ± 8.1	56.7 ± 8.2	56.4 ± 8.0
Current smoker	50,664 (10.5)	27,391 (12.4)	23,273 (8.9)
Blood pressure, systolic, mm Hg	139.8 ± 19.7	142.8 ± 18.5	137.3 ± 20.3
Diabetes diagnosed by doctor	24,920 (5.2)	15,336 (7.0)	9,887 (4.5)
Hypertension	254,564 (52.7)	133,013 (60.4)	121,533 (46.3)
Family history, first-degree relative	206,363 (42.8)	87,946 (39.9)	118,417 (45.1)
High cholesterol	65,829 (13.6)	37,801 (17.2)	28,028 (10.7)
Prevalent CAD events before age 75 yrs	9,729 (2.0)	7950 (3.6)	1779 (0.7)
Incident CAD events before age 75 yrs	12,513 (2.6)	9320 (4.2)	3193 (1.2)
On blood-pressure lowering medication	99,454 (20.6)	53,535 (24.3)	45,939 (17.5)
On lipid-lowering medication	82,493 (17.1)	49,459 (22.5)	33,028 (12.6)
Follow-up time, yrs	6.2 ± 2.1	5.9 ± 2.6	6.4 ± 1.4

Values are mean ± SD or n (%). CAD = coronary artery disease.

**Figure 2:**
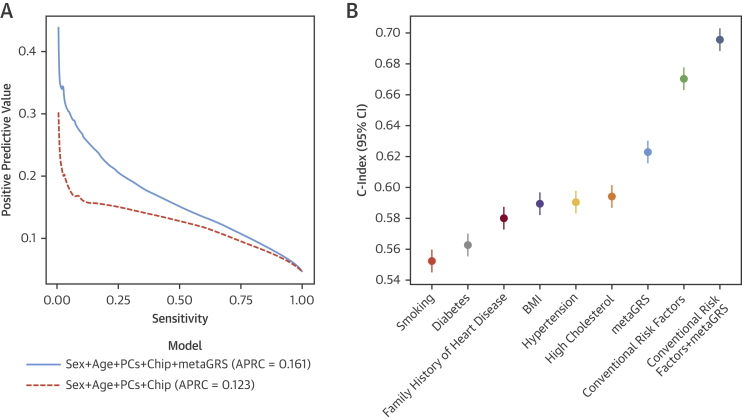
Predictive Measures of CAD Using the metaGRS and Conventional Risk Factors **(A)** Positive predictive values versus sensitivity for the reference model (sex + age + array + 10 genetic PCs) and when adding the metaGRS to the model for all CAD in the UKB testing set. **(B)** C-index for sex-stratified age-as-time-scale Cox regression of incident CAD for conventional risk factors individually and in combination with the metaGRS, including genotyping array and 10 genetic PCs as covariates. APRC = area under the precision-recall curve; other abbreviations as in Figure 1.

In the external UKB validation set, the metaGRS was accurate at classifying CAD cases versus noncases, with an area under the receiver-operating curve of 0.79 (+2.8% over the reference logistic model consisting of sex, age at assessment, genotyping array, and 10 PCs). The metaGRS offered greater positive predictive value at any given sensitivity and, thus, greater area under the precision-recall curve (recall is also known as sensitivity) compared with the reference model (0.161 vs. 0.123) (Figure 2A). The distributions of the metaGRS amongst prevalent CAD cases, incident CAD cases, and non-CAD cases were each approximately Gaussian and revealed a trend of increasing genomic risk (Online Figure 2), with prevalent cases more easily differentiable, as they likely comprise individuals who are at higher genomic risk and have thus had earlier CAD events.

In sex-stratified Cox regression models for all CAD (prevalent and incident), the metaGRS had an HR of 1.71 (95% confidence interval [CI]: 1.68 to 1.73) per SD of metaGRS (p < 0.0001) (Figure 1). The metaGRS was significantly but weakly associated with body mass index (BMI) at assessment (0.0044 log[kg/m^2^] per SD; 95% CI: 0.0039 to 0.0049; p < 0.0001), diagnosed diabetes (odds ratio [OR]: 1.14 per SD; 95% CI: 1.13 to 1.16; p < 0.0001), hypertension at assessment (OR: 1.19 per SD; 95% CI: 1.18 to 1.20; p < 0.0001), current smoking at assessment (OR: 1.06 per SD; 95% CI: 1.04 to 1.07; p < 0.0001), family history of heart disease (OR: 1.21 per SD; 95% CI: 1.199 to 1.214; p < 0.0001), and self-reported high cholesterol at/before assessment (OR: 1.27 per SD; 95% CI: 1.26 to 1.28; p < 0.0001). No evidence for competing risk effects was observed (Online Figure 3). In Cox regression of incident CAD (Figure 2B), models based on the metaGRS had higher C-index (C = 0.623; 95% CI: 0.615 to 0.630) than any of the individual conventional risk factors, with the second-best factor being self-reported high cholesterol at assessment (C = 0.594; 95% CI: 0.587 to 0.601). A model combining the 6 conventional risk factors had only slightly better performance (C = 0.670; 95% CI: 0.663 to 0.678) than the metaGRS individually. Combining the metaGRS with all 6 conventional risk factors led to a model with C-index of 0.696 (95% CI: 0.688 to 0.703), an increase of 2.6% over the model consisting of the 6 conventional risk factors. When adjusting for conventional risk factors, only incident CAD cases could be considered; however, the HR for metaGRS was only modestly attenuated (HR: 1.58 per SD; 95% CI: 1.55 to 1.61 not adjusting for risk factors; HR: 1.55 per SD; 95% CI: 1.52 to 1.58 adjusting for family history; HR: 1.48 per SD; 95% CI: 1.45 to 1.51 after adjustment for 6 other risk factors).

**Figure 3:**
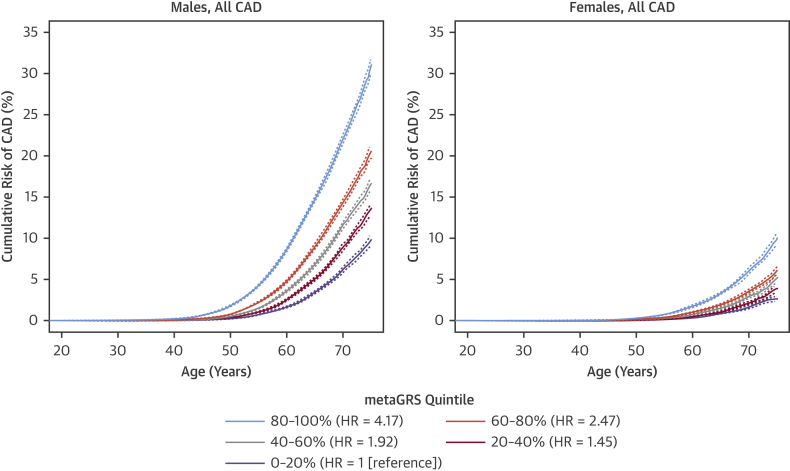
Cumulative Risk of CAD by Quintiles of metaGRS in Men and Women **Dotted lines** represent 95% confidence intervals. For subgroup sample sizes, see Online Table 1. HR = hazard ratio; other abbreviations as in Figure 1.

To investigate the potential role of the metaGRS in earlier life genetic screening, we compared the sex-stratified cumulative incidence of CAD across quintiles of the metaGRS (Figure 3). In UKB men, we observed that CAD risk in the highest metaGRS quintile began exponentially increasing shortly after age 40 years, reaching a threshold of 10% cumulative risk by 61 years of age (Figure 3). By comparison, CAD risk for men in the lowest metaGRS quintile did not begin increasing until age 50 years, and on average, did not reach 10% by the censoring age of 75 years. In UKB women, the metaGRS results were similar but delayed given the lower absolute CAD risk overall compared with men. For women in the highest metaGRS quintile, CAD risk began increasing at age 49 years and reached 10% at age 75 years, whereas women in the lowest metaGRS quintile were at extremely low levels of risk, reaching 2.5% CAD risk by the censoring age of 75 years. There was no evidence for a statistical interaction of the metaGRS with sex. Overall, on average, UKB individuals in the top metaGRS quintile were at 4.17-fold (95% CI: 3.97- to 4.38-fold) higher hazard of CAD than those in the bottom metaGRS quintile (Figure 3).

**Figure 4:**
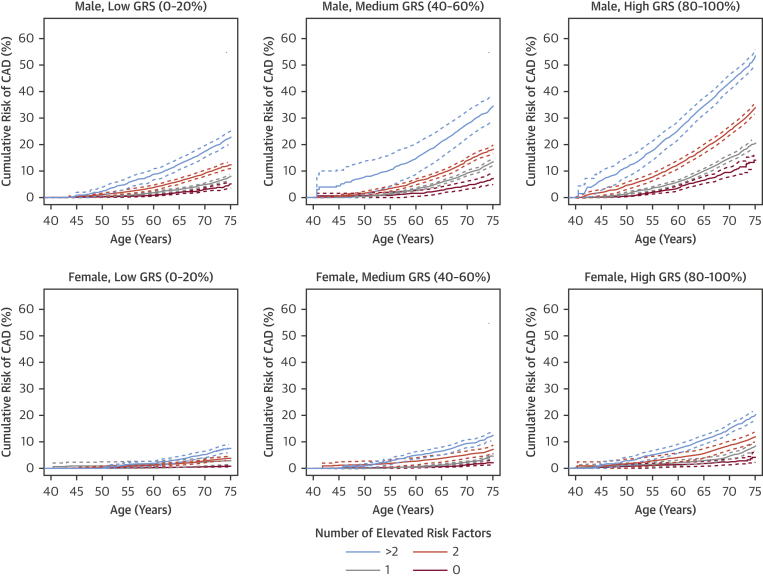
Cumulative Risk of Incident CAD for Increasing Numbers of Conventional Risk Factors Stratified by metaGRS Quintile **Dotted lines** represent 95% CIs. GRS = genomic risk score; HR = hazard ratio; other abbreviations as in Figure 1.

We next assessed the differences in incident CAD risk across metaGRS quintiles when combined with conventional risk factors (current smoking, diagnosed diabetes, high blood pressure, high BMI, family history of heart disease, and high cholesterol) individually (Online Figures 4 to 9) or as an unweighted score, the number (0 to 6) of conventional risk factors per individual (Figure 4). Broadly, the patterns were similar across all of the analyses. Genomic risk and lifestyle/clinical factors combined to be associated with higher risk in both men and women; however, in most instances, this was additive rather than interactive. In Cox regression models of incident CAD, adjusting for current smoking, diagnosed diabetes, hypertension, log BMI, family history, high cholesterol, genotyping array, and 10 genetic PCs, there was no strong evidence of statistical interactions between the metaGRS and diabetes (p = 0.074 for interaction), smoking (p = 0.13 for interaction), hypertension (p = 0.93 for interaction), family history (p = 0.51 for interaction), or high cholesterol (p = 0.14 for interaction), but there was some evidence for interaction with log BMI (HR: 0.85; 95% CI: 0.76 to 0.95; p = 0.0052). From a clinical perspective, it was notable that men in the highest metaGRS quintile who had no conventional risk factors still reached 10% cumulative incidence of CAD by age 69 years, with a similar cumulative incidence as men in the lowest metaGRS quintile who had 2 elevated conventional risk factors (Figure 4). Men in the highest metaGRS quintile and with 3 or more conventional risk factors were at extremely high levels of CAD risk, reaching the 10% threshold by age 48 years. Approximately 79% of women did not reach 10% CAD risk before age 75 years, even if they had 2 conventional risk factors, due to compensation by low or moderate metaGRS risk. Even amongst women in the highest metaGRS quintile, only those with 2 or more conventional risk factors achieved 10% risk before age 75 years (Figure 4).

**Figure 5:**
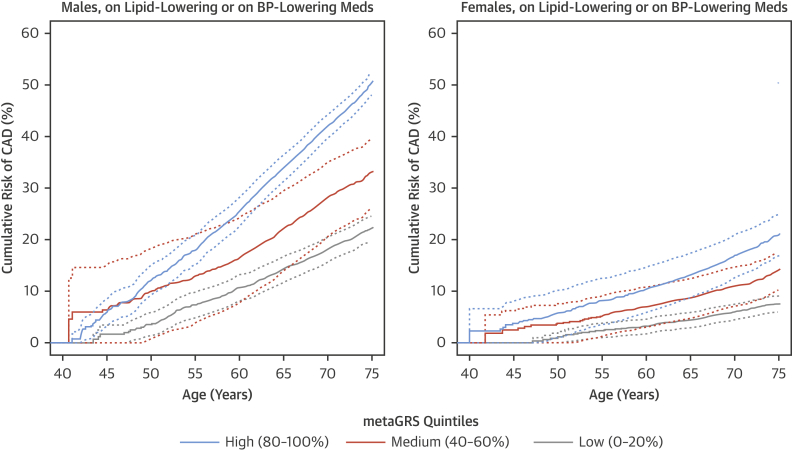
Cumulative Risk of Incident CAD Within Individuals on Lipid-Lowering or BP-Lowering Medication at Assessment **Dotted lines** represent 95% CIs. BP = blood pressure. Abbreviations as in Figure 1.

To assess the impact of use of treatments (lipid-lowering and antihypertensive medication) that have been proven to lower CAD risk on the performance of the metaGRS, we analyzed the association of the metaGRS with incident CAD in those taking 1 or both of these classes of drugs at baseline. The HRs for each SD in GRS were reduced but not negated by these therapies, with HRs of 1.44 (95% CI: 1.40 to 1.48), 1.46 (95% CI: 1.42 to 1.50), and 1.42 (95% CI: 1.37 to 1.47) for those individuals on lipid-lowering, antihypertensive, or both treatments, respectively. Accordingly, the HRs between those in the top versus bottom metaGRS quintiles were also reduced but remained substantial, with HRs of 2.71 (95% CI: 2.47 to 2.98), 2.81 (95% CI: 2.56 to 3.09), and 2.55 (95% CI: 2.28 to 2.86), for those individuals on lipid-lowering, antihypertensive, or both treatments, respectively (Figure 5).

## Discussion

In an analysis of almost 500,000 people in a prospective nationwide cohort study, we evaluated a combined genomic risk score (metaGRS) built from summary statistics of the largest previous genome-wide association studies of CAD (Central Illustration). We report a series of findings that substantially advance the concept of using genomic information to help stratify individuals for CAD risk in general populations, an approach that leverages the fixed nature of germline DNA over the life course to anticipate different lifelong trajectories of CAD risk.

**Central Illustration:**
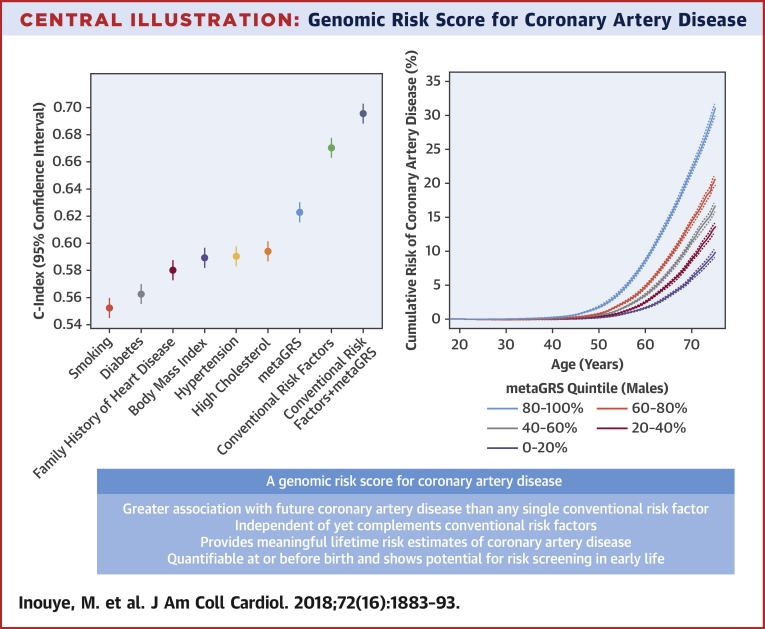
Genomic Risk Score for Coronary Artery Disease The genomic score provides potential for risk screening early in life as well as complements conventional risk factors for coronary artery disease.

First, our metaGRS achieved greater risk discrimination than previously published genomic risk scores based on selected SNPs 3, 4, 5, 6, 7, 8, 9. For example, we found metaGRS had a greater HR and positive predictive value at any given sensitivity, as well as a 4-fold HR for CAD in a comparison of individuals in the top versus bottom one-fifth of the risk score distribution.

Second, we found that the predictive ability of the metaGRS was largely independent of established risk factors for CAD, implying that genetic information complements (rather than replaces) conventional risk factors. As our data have suggested that higher genetic risk can at least partly be attenuated by lipid-lowering and/or antihypertensive therapies, it implies that individuals at high genetic risk may gain the most from early initiation of these therapies and, therefore, constitute a subpopulation for which primary prevention may be particularly cost-effective (7). However, as our results have suggested that the metaGRS predicts CAD risk even among individuals taking CAD therapies at baseline, it also underscores the need to develop new therapies to address residual disease risk.

Third, we found that the metaGRS identified individuals who are at high risk of premature CAD as well as those unlikely ever to reach a life-long risk level requiring intervention. For example, our findings have suggested that because men in the highest metaGRS quintile are at such high risk, they are likely to benefit from more intensive preventative interventions regardless of levels of traditional clinical risk factors. By contrast, the present findings suggest that about 80% of women in general populations (i.e., those not in the top 20% of the metaGRS) may not benefit from intensive preventive interventions, in the absence of other compelling indications, before age 75 years. This finding underscores the potential value of using genomic information to optimize use of scarce resources for disease prevention; however, further health economic studies would be necessary.

Although applied health studies will be needed to evaluate properly the clinical utility of CAD genomic risk scores, elements of potential clinical implementation can now be foreseen. For example, genome-wide array genotyping has a 1-time cost (approximately US$50 at current prices) and can be used to calculate updated genomic risk scores for CAD as further, more powerful association data emerge. Indeed, data from a genome-wide genotyping array can be utilized to calculate GRS for a wide range of common diseases. To calculate genomic risk for individuals, simple algorithms can draw on information from such arrays, as well as from large reference groups from similar populations, such as UK Biobank. In translating genomic risk scores, standardization in assay and data processing will be necessary but achievable, including in imputation (e.g., reference panel and quality control) and handling of population stratification (e.g., using a population-specific GRS distribution and/or adjustment of GRS directly). We have made the metaGRS algorithm freely available (21) to facilitate development and translation of the concept of genomic risk as an early screening tool.

### Study limitations

First, while previous studies have shown the added value of a GRS to clinical risk scores, such as Framingham Risk Score and ACC/AHA13 Risk Score (3), UK Biobank does not yet have measurements of lipids and other biochemical factors available; thus, relationships of the metaGRS with lipids or traditional clinical risk scores (e.g., Framingham Risk Score, QRISK, and so on) could not be assessed. Second, the UK Biobank has a minimum enrollment age of 40 years, and participants have been shown to be healthier than the UK general population 22, 23; thus, our study may have underestimated population-level lifetime CAD risk. Third, people of non-European ancestry make up a small proportion (<5%) of the UK Biobank, suggesting the need for studies in people of other ancestries. Similarly, future studies that externally validate the metaGRS in large multiethnic cohorts would maximize generalizability and minimize risk of overfitting to any single dataset or population (24). Fourth, current GWAS sample sizes and imputation efficiencies are also limiting in that they introduce noise into GRS estimates. Our meta-score approach here addresses this to some extent; however, future large-scale cohorts will offer more powerful genomic scores. Last, despite the metaGRS showing substantial CAD risk discrimination in individuals already on medication, we were also unable to assess the effect of medication versus nonmedication in individuals who are at high metaGRS risk, as without blind randomization, this analysis would be susceptible to reverse causation, with those on medication likely already at higher CAD risk.

## Conclusions

The genomic score developed and evaluated in the present study strengthens the concept of using genomic information to stratify individuals for CAD risk in general populations and demonstrates the potential for genomic screening in early life to complement conventional risk prediction.

Perspectives **COMPETENCY IN MEDICAL KNOWLEDGE:** Genetically determined risk of CAD is largely independent of conventional risk factors, such as lipids, blood pressure, and smoking. As a predictor of CAD, a meta-score (metaGRS) derived from a U.K. biobank outperformed other genetic risk scores and individual conventional risk factors, even in patients treated with lipid-lowering or antihypertensive medications.

**TRANSLATIONAL OUTLOOK:** Future studies should determine how best to employ genetically predicted risk for primary prevention of CAD.
