# Supplementary material for: Genomic Risk Prediction of Coronary Artery Disease in 480,000 Adults: Implications for Primary Prevention
Source: J Am Coll Cardiol. 2018 Oct 16;72(16):1883–93. doi: 10.1016/j.jacc.2018.07.079 (PMC6176870; doi:10.1016/j.jacc.2018.07.079)
Supplement: Online Data [file mmc1.docx]

**Supplemental Information**

**Data processing and quality control**

Only autosomal genetic variants imputed using the HRC panel and which had MAF >0.1% were included in our analyses, totaling 14.5 million variants. We converted the imputed dosages to PLINK [1] genotype calls with minimum probability 0.9 (otherwise the call was set to missing; we removed variants with >1% missingness across individuals). To control for population structure, we utilized the genetic principal components (PCs) as given by the UKB [2].

Two QC schemes were used. For the GRS46K and FDR202 genetic risk scores (defined below), we kept variants with MAF >0.1%, however filtering by HWE and imputation quality (INFO) was not employed as this led to fewer variants mapping to these scores and thus reductions in predictive power. For the 1000Genomes CAD score, we included variants with impute2 INFO >0.01 and HWE *P*>10^-12^. A lenient HWE threshold was used because HWE was computed over all individuals without regard to population structure, thus variants that deviation from HWE at a stringent threshold may be needlessly excluded as they may not be indicative of genotyping error. A lenient INFO threshold (e.g. relative to the INFO >0.4 used in Nikpay et al [3]) was employed as the UKB has a relatively large sample size, and an analysis of variants with INFO=0.001 in the UKB has equivalent statistical power to an analysis of 0.001 x 500,000 = 500 individuals; thus genetic variants with low INFO score can still offer improvements in predictive accuracy. Similarly, bias in odds ratios arising from genotyping or imputation error has the effect only of reducing the predictive accuracy of a GRS, but this reduction is less than would occur by omitting the marker entirely. After the above quality control procedures were implemented, 14,516,436 autosomal markers were available for subsequent analyses. For the 1000Genomes CAD score, we only used markers found in both the UKB data and the 1000Genomes CAD summary statistics, resulting in 5,176,852 autosomal variants. In the UKB, 485,629 individuals had matching genetic data and CAD outcome data. We removed individuals with (i) diagnoses in one of ICD9 414.1, ICD10 I25.0, I25.3, I25.4 or (ii) CAD event but no known age or date of CAD. There was no evidence of differential missingness in genetic data between CAD and non-CAD individuals. The final dataset consisted of n=485,629 individuals, including 22,242 CAD events and 460,387 non-cases.

**Construction of the metaGRS for coronary artery disease**

To construct a meta genomic risk score (metaGRS) for CAD, we followed a meta-analysis strategy using the largest CARDIoGRAMplusC4D genome-wide association analysis without UKB data [3] and the CARDIoGRAMplusC4D Metabochip analysis, which focused on variants and regions known or thought to be associated with cardiometabolic phenotypes [4]. The GRS46K (46,000 genetic variants, excluding ~3,000 A/T and C/G SNPs) was constructed previously [5] using linkage disequilibrium (LD) thinning of 79,000 SNPs from the CARDIoGRAMplusC4D Metabochip meta-analysis [4], while the FDR202 and '1000Genomes' CAD GRS derived from the CARDIoGRAMplusC4D genome-wide association analysis, are described below. The three GRSs all provide an imperfect measure of an individual’s genomic risk of developing CAD, due to incomplete and targeted coverage of the genome, finite sample estimates of the marginal effect sizes for each genetic variant, and genotyping or imputation uncertainty. Since it is well known that a risk factor measured with error can attenuate the association between the risk factor and disease occurrence (regression dilution bias [6]), we reasoned that a ‘meta score’, the weighted average of the three standardized genetic risk scores, would provide a more precise estimate of an individual’s genomic risk of developing CAD.

To create the metaGRS, we used the meta-analysis summary statistics [3], consisting of dbSNP rsid, risk allele, and effect size (log odds ratio). We used the existing GRS46K [5] and FDR202 [3] scores. The GRS46K was previously derived using LD thinning of the CARDIoGRAMplusC4D Metabochip summary statistics [4] (with subsequent removal of 3,000 A/T or G/C SNPs). The FDR202 score utilizes the set of 202 independent SNPs and indels identified at false discovery rate (FDR) <0.05 together with their corresponding weights (log odds) in the CARDIoGRAMplusC4D 1000Genomes meta-analysis [3]. For the GRS46K, we could map 45,810 variants (98%) in the UKB imputed dataset; for the FDR202, 198 variants or proxies thereof mapped (98%; for proxies, minimum *r*^2^ in 1000Genomes CEU+GBR was 0.68, the median *r*^2^ was 1.0).

Each score *s* is the sum of the minor allele dosages of each variant multiplied by its marginal effect size:

$$s_{i}=\sum_{j=1}^{m} x_{ij}\beta_{j}$$

where $x_{ij}\in\left\{ 0, 1, 2 \right\}$ is the count of the minor allele for the jth variant in the ith individual and *β* _j_ is the marginal effect size (log odds ratio per dosage of minor allele) for the j^th^ variant.

To derive a new genomic risk score ('1000Genomes') based on the CARDIoGRAMplusC4D 1000Genomes-imputed GWAS summary statistics [3], we split the UKB data into a training set (n=3000) and a validation set (n=482,629). For the training set, we randomly selected 1000 prevalent CAD cases and 2000 non-CAD individuals. We then used PLINK random LD pruning to create different scores, based on SNP sets with varying levels of LD and corresponding CARDIoGRAMplusC4D summary statistics, and evaluated their performance on the n=3000 training set, in terms of hazard ratio (HR) per standard deviation (s.d.) of the score (age-as-time-scale Cox regression, stratified by sex and adjusting for BiLEVE/UKB genotyping array and 10 genetic PCs). The score with the highest HR corresponded to the *r*^2^ thinning threshold of 0.9 and consisted of ~1.7 million variants.

The correlation between the three GRSs was moderate (Pearson’s correlation *r*=0.11 for FDR202-GRS46K, *r*=0.19 for FDR202-1000Genomes, *r*=0.27 for GRS46K-1000Genomes), indicating partial but imperfect overlap of the genetic signals captured by each, likely due to shared genetic loci, LD, and partial overlap of individuals in the cohorts used for deriving these summary statistics [3, 4]. Such correlation is accounted for in the weighting below.

We derived a meta score (‘metaGRS’), consisting of a weighted average of the standardized scores

$$\mathrm{GRS}_{i}^{\mathrm{meta}}=\frac{\beta_{1}Z_{i1}+\beta_{2}Z_{i2}+\beta_{3}Z_{i3}}{\sqrt{\beta_{1}^{2}+\beta_{2}^{2}+\beta_{3}^{2}+2\beta_{1}\beta_{2}\rho_{1,2}+2\beta_{1}\beta_{3}\rho_{1,3}+2\beta_{2}\beta_{3}\rho_{2,3}}}$$

where $Z_{i1},Z_{i2},Z_{i3}$are the (zero-mean and unit-variance standardised) GRS46K, FDR202, and 1000Genomes CAD risk scores for the *i*th individual, respectively, $\beta_{1}$,$\beta_{2}, \beta_{3}$ are the univariate log HRs for each score (estimated using Cox regression in the training set), and $\rho_{i,j}$ is the Pearson correlation between the *i*th and *j*th scores (in the training set). The univariate log HRs were 0.1278, 0.2359 and 0.2400 per 1-s.d. for the GRS46K, FDR202, and 1000Genomes CAD scores, respectively. In terms of SNP-level genotype, the meta score above can be expressed in terms of the weighted sum over all *m* = 1,745,180 SNPs (the union of the SNPs in the three scores, and ignoring constant terms),

$$\mathrm{GRS}_{i}^{\mathrm{meta}}\propto\sum_{j=1}^{m} x_{ij}\left( \frac{\beta_{1}}{\sigma_{1}}\alpha_{j1}+\frac{\beta_{2}}{\sigma_{2}}\alpha_{j2}+\frac{\beta_{3}}{\sigma_{3}}\alpha_{j3} \right),$$

where $\sigma_{1}, \sigma_{2},\sigma_{3}$ are the empirical s.d.'s of the scores (GRS46K, FDR202, and 1000Genomes CAD) in the training data, $\alpha_{j1},\alpha_{j2},\alpha_{j3}$ are the SNP effect sizes (log odds ratios from the published summary statistics) for the *j*th SNP in each of the three scores, respectively, and $x_{ij}$ is the genotype for the *i*th individual’s *j*th SNP. A SNP’s effect size $\alpha_{jk}$ was considered to be zero for the *k*th score if the SNP was not included in that score.

SNP heritability explained by the 1.7 million SNPs was calculated by residual maximum likelihood (REML) in BOLT-LMM [7], and converted to the liability-threshold scale assuming a CAD population prevalence of 5% and a within-study prevalence of CAD 4.61%.

**Supplemental References**

1. Chang, C.C., et al., *Second-generation PLINK: rising to the challenge of larger and richer datasets*. Gigascience, 2015. 4: p. 7.

2. Bycroft, C., et al., *Genome-wide genetic data on~ 500,000 UK Biobank participants*. bioRxiv, 2017: p. 166298.

3. Nikpay, M., et al., *A comprehensive 1,000 Genomes-based genome-wide association meta-analysis of coronary artery disease*. Nat Genet, 2015. 47(10): p. 1121-1130.

4. CARDIoGRAMplusC4D Consortium, et al., *Large-scale association analysis identifies new risk loci for coronary artery disease*. Nat Genet, 2013. 45(1): p. 25-33.

5. Abraham, G., et al., *Genomic prediction of coronary heart disease*. Eur Heart J, 2016. **37**(43): p. 3267-3278.

6. MacMahon, S., et al., *Blood pressure, stroke, and coronary heart disease. Part 1, Prolonged differences in blood pressure: prospective observational studies corrected for the regression dilution bias*. Lancet, 1990. 335(8692): p. 765-74.

7. Loh, P.R., et al., *Efficient Bayesian mixed-model analysis increases association power in large cohorts*. Nat Genet, 2015. 47: p. 284-290.

8. Lee, S.H., et al., *Estimating missing heritability for disease from genome-wide association studies.* Am J Hum Genet, 2011. **88**(3): p. 294-305

**Supplemental Table 1: Sample sizes for metaGRS quintile subgroups**

Males

|  | Number at risk (cumulative number of censored) | | | | |
| --- | --- | --- | --- | --- | --- |
| metaGRS quintile | Age 30 | Age 40 | Age 50 | Age 60 | Age 70 |
| 0-20% | 43963 (0) | 43960 (0) | 40712 (3298) | 29121 (14474) | 10858 (31933) |
| 20-40% | 44514 (0) | 44503 (0) | 41076 (3435) | 29124 (14739) | 10636 (31956) |
| 40-60% | 43937 (0) | 43925 (0) | 40480 (3376) | 28256 (14618) | 10113 (31253) |
| 60-80% | 44035 (0) | 44007 (0) | 40304 (3562) | 27555 (15038) | 9624 (31060) |
| 80-100% | 43833 (0) | 43761 (0) | 39102 (4181) | 25023 (16039) | 8062 (30446) |

Females

|  | Number at risk (cumulative number of censored) | | | | |
| --- | --- | --- | --- | --- | --- |
| metaGRS quintile | Age 30 | Age 40 | Age 50 | Age 60 | Age 70 |
| 0-20% | 52563 (0) | 52563 (0) | 48896 (3831) | 34592 (18091) | 12063 (40441) |
| 20-40% | 52012 (0) | 52010 (0) | 48328 (3858) | 33651 (18423) | 11686 (40117) |
| 40-60% | 52589 (0) | 52584 (0) | 48723 (4025) | 33774 (18757) | 11736 (40394) |
| 60-80% | 52491 (0) | 52486 (0) | 48436 (4231) | 33364 (19001) | 11424 (40439) |
| 80-100% | 52692 (0) | 52682 (0) | 48194 (4571) | 31839 (20452) | 10196 (41202) |

**Supplemental Figure 1: Flowchart of the study**

**Supplemental Figure 2**: **Density plots of the metaGRS within the UKB external validation set,** comparing prevalent CAD (n=9,729) with incident CAD before 75y (n=12,513) and non-cases (n= 460,387).

**
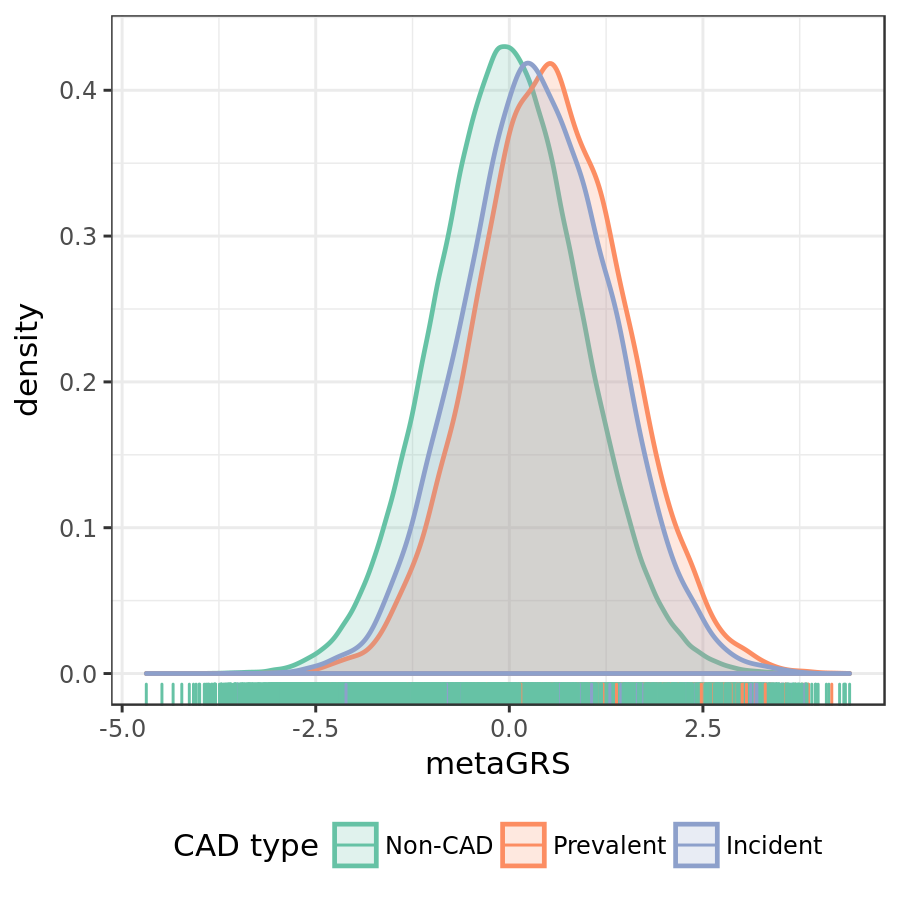
**

**Supplemental Figure 3**: Comparison of Kaplan-Meier estimates of cumulative incidence of CAD (censored at 75y) with the Aalen-Johansen estimates of competing risk (using three states: CAD, non-CAD death, censored), in the UKB testing dataset. Non-CAD deaths n=11,182.

**Supplemental Figure 4: Cumulative incidence of CAD for current smokers vs non-smokers at baseline stratified by metaGRS quintiles**

**Supplemental Figure 5:** **Cumulative incidence of CAD for individuals with/without diagnosed diabetes at baseline, stratified by metaGRS quintiles**

**Supplemental Figure 6:** **Cumulative incidence of CAD for individuals with/without hypertension at baseline, stratified by metaGRS quintiles**

**Supplemental Figure 7:** **Cumulative incidence of CAD for individuals with body mass index (BMI) <30 or ≥30 at baseline, stratified by metaGRS quintiles**

**Supplemental Figure 8:** **Cumulative incidence of CAD for individuals with 1^st^-degree family history of heart disease at baseline, stratified by metaGRS quintiles**

**Supplemental Figure 9:** **Cumulative incidence of CAD for individuals with known high cholesterol at or before baseline, stratified by metaGRS quintiles**
